# Supplementary material for: Real-time multi-task diffractive deep neural networks via hardware-software co-design
Source: Sci Rep. 2021 May 26;11:11013. doi: 10.1038/s41598-021-90221-7 (PMC8155121; doi:10.1038/s41598-021-90221-7)
Supplement: Supplementary file 1 — Supplementary Information. [file 41598_2021_90221_MOESM1_ESM.pdf]

Real-time Multi-Task Diffractive Deep Neural Networks via Hardware-Software Co-design

Yingjie Li,[1] Ruiyang Chen,[1] Berardi Sensale Rodriguez,[1], Weilu Gao,[1] Cunxi Yu[1*]

[1]Electrical and Computer Engineering Department, University of Utah
50 S Central Campus Road, Salt Lake City, Utah, USA, 84112

*E-mail: cunxi.yu@utah.edu

Supplementary materials

Derivation of D^{2}NN The forward propagation follows the model described in [17], where the light propagation consists of free space propagation of diffractive light and transparency phase modulation, as shown in Figure 6 (in the main file). The phase modulation follows the direct multiplication of input wavefunction and phase function of masks, and free space propagation features the interconnects (addition of wavefunctions) between layers. Here, we details the treatment of free space propagation implemented in our model.

In experimental process, as shown in Figure 6 (in the main file), the input light passes through a set of diffractive layers and then is collected by output detectors. When light is incident on one diffractive layer, according to *Huygens-Fresnel* principle , each point on the output of the diffractive layer can be seen as a secondary point source emitting spherical waves and the output wave is the sum of all these spherical waves. It means the inputs of every point on l-th layer can be seen as the sum of outputs from all the points on $(l-1)$th layer. This features the interconnects in a neural network and each point acts as a neuron.

The free-space propagation relation is

$$g(x, y) = f(x, y)h(x, y), \tag{1}$$

where $f(x, y)$ is input function and $h(x, y)$ is the impulse response function of free space. Under Fresnel's ap-

proximation, the impulse response function is:

$$h(x,y)\approx\frac{j}{\lambda z}\text{exp}(-jkz)\text{exp}(-j\pi\frac{x^2+y^2}{\lambda z}) \tag{2}$$

Thus, the input at point (x, y) on l-th layer can be written as the sum of all the outputs at $(l-1)$-th layer:

$$g_l(x,y)=\iint f_{l-1}(x',y')h(x-x',y-y')dx'dy', \tag{3}$$

where

$$h(x-x',y-y')\approx\frac{j}{\lambda d}\text{exp}(-jkd)\text{exp}(-j\pi\frac{(x-x')^2+(y-y')^2}{\lambda d}) \tag{4}$$

and d is the distance between layers. f_{l-1} is the output wavefunction of points on $(l-1)$th plane and also the input wavefunction of free space propagation, g_l is the output function of free-space propagation and also input for the phase mask at l-th plane.

In simulation process, the convolution of Eq. 3 is complicated for implementation and training. To speed up and simplify the training process, especially under `Pytorch` framework, Fourier transform to spatial frequency domain is employed. Specifically, the free space propagation output wavefunction (3) is in convolution form $f_{l-1} * h$. By convolution theorem, the Fourier transform of the convolution is the product of Fourier transforms of f_{1-1} and h:

$$\mathcal{F}(g_l(x,y))=\mathcal{F}(f_{l-1}(x,y))\mathcal{F}(h(x,y)) \tag{5}$$

$$U_l(f_x,f_y)=F_{l-1}(f_x,f_y)H(f_x,f_y) \tag{6}$$

The Fourier transform of h is

$$H(f_x,f_y)=\mathcal{F}(h(x,y))=\text{exp}(jkd)\text{exp}(j\pi\lambda z(f_x+f_y)^2) \tag{7}$$

This is the free space transfer function.

As shown in Figure 1, for the free space propagation between layers, the signal is firstly converted to spatial frequency domain (f_x, f_y) through fast Fourier transformation (FFT). According to Equation (6), the output of free

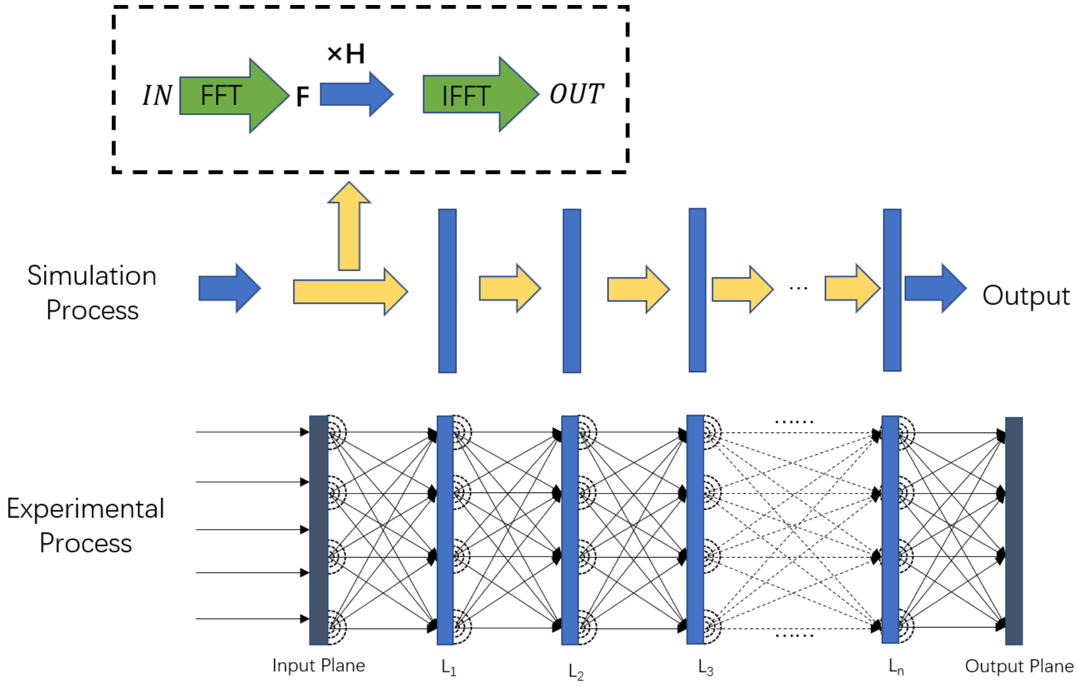

Figure 1: Simulation and experimental process

space propagation is simply the product of the input FFT signal and free space transfer function H. After the free space propagation, the FFT signal is converted back to spatial domain (x, y) through inverse Fourier transformation (IFFT) and the obtained signal is modulated through the phase mask.

Beam splitter noise evaluation Similar to noise evaluation shown in Figure 4 (in the main file), we evaluate the prediction performance under two different noises, i.e., beam splitter noise with detector noise and beam splitter noise with device variations. The noise modeling has been discussed in Section Methods. Figure 2 (in this SI file) present the prediction accuracy of both tasks under these two noise set ups. We can see that the splitter noise does not have significant impacts on the accuracy compared to other system noise. As shown in Figure 2(a)–(b) (in this SI file), the accuracy degradation is basically dominated by the detector noise; and as shown in Figure 2(c)–(d) (in this SI file), the accuracy degradation is basically dominated by the device variation.

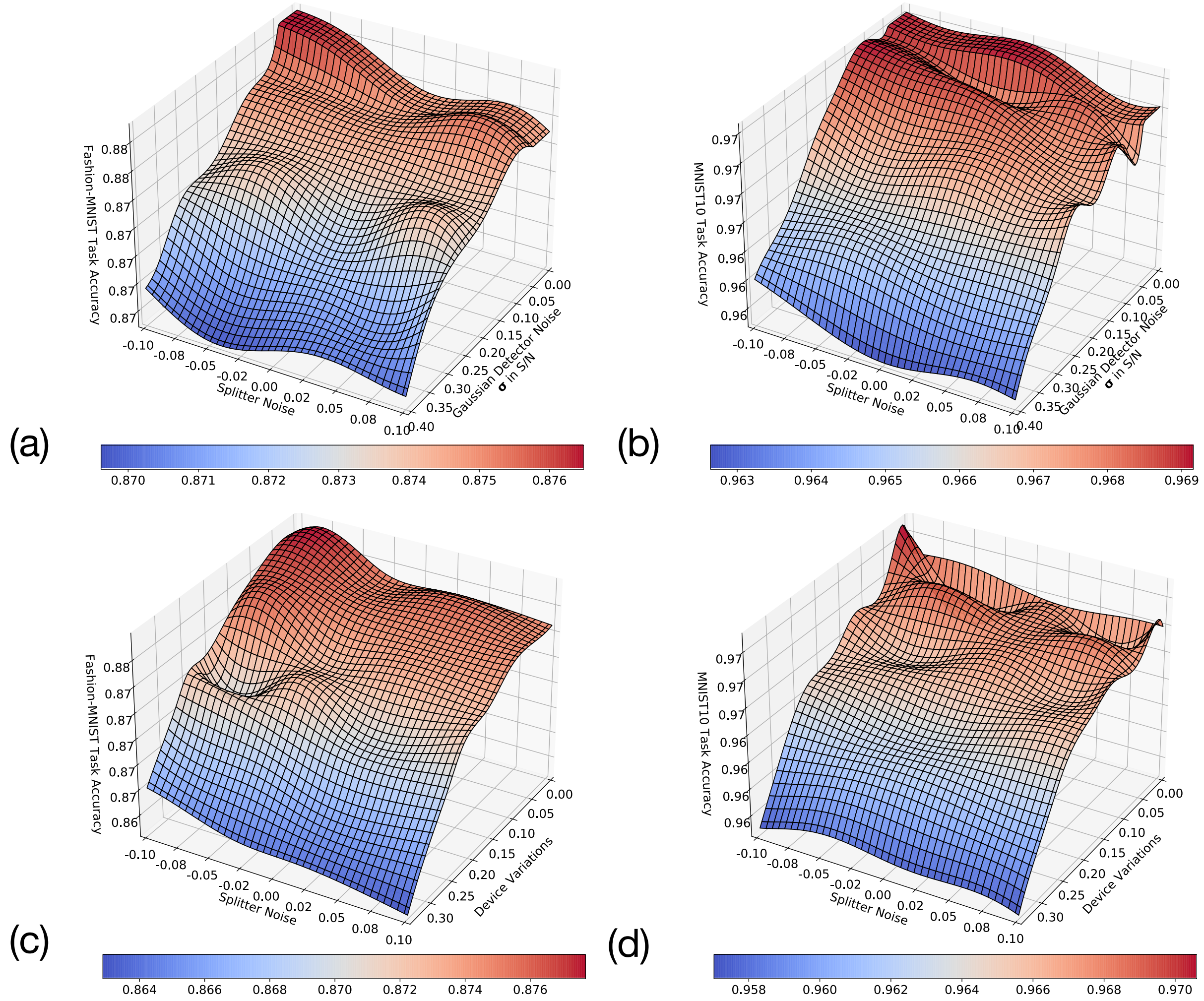

Figure 2: **Evaluation of 50-50 beam splitter noise with device variations and detector noise.** (a)–(b) Prediction performance evaluation under Gaussian detector noise with σ shown in S/N (Signal to Noise) $\in [0, 0.2]$ and beam splitter noise $\in [-0.1, 0.1]$, with accuracy of MNIST shown in (a) and accuracy of Fashion-MNIST shown in (b). (c)–(d) Prediction performance evaluation under Gaussian device variation noise with $\sigma \in [0, 0.15]$, and beam splitter noise $\in [-0.1, 0.1]$, with accuracy of MNIST shown in (c) and accuracy of Fashion-MNIST shown in (d).
